# Synchronous Soleus and Reverse Sural Flap for Large Soft Tissue Defect Reconstruction of Leg

**Published:** 2018-01

**Authors:** Seyed Esmail Hassanpour, Masoud Yavari, Amir Reza Motabar

**Affiliations:** Department of Plastic Surgery, School of Medicine, Shahid Beheshti University of Medical Sciences, Tehran, Iran

**Keywords:** Wound, Soleus, Sural, Flap, Leg, Soft tissue, Reconstruction

## Abstract

**BACKGROUND:**

Extended Soft tissue defect of leg including middle and distal parts always has been a challenge for many plastic surgeons and also a frustrated problem for patients and families. To introduce the use of the soleus muscle and reverse sural flaps as synchronous surgical treatment alternative of the leg bone exposure with large soft tissue defect, this study was conducted.

**METHODS:**

The medical records of patients undergoing transposition of the soleus muscle for treating exposed bone in the leg and simultaneous sural flap were retrospectively analyzed from January 2009 to July 2014, while gathering information on the used muscle was to cover the lesion.

**RESULTS:**

Twelve patients with varying ages between 22 and 58 years (10 males and 2 females) were enrolled. The main initial injury was trauma (84.8%), consisting of tibia and/or fibula fractures. Only 1 patient developed insignificant distal flap necrosis who was treated subsequently with surgical debridement and flap re-advancement.

**CONCLUSION:**

The treatment of bone exposure with local muscle flaps (soleus and sural) enables obtaining satisfactory results in covering of exposed structures, favoring local vascularization and improving the initial injury. It offers the advantage of providing a treatment in only one surgical procedure, an earlier recovery and reduced hospital stay. Sometimes, this method may be applied instead of free tissue transfer.

## INTRODUCTION

Extended Soft tissue defect of leg include middle and distal parts have always been a challenge for many plastic surgeons and also frustrated problems for patients and families. The main goals of soft tissue reconstruction is to cover exposed bone, tendon, or hardware. Small and moderate-size wounds of the leg may be better served by the use of local and regional flaps,^1^ but in large defects, these flaps are not available and more complex and different flap designs are required.^2^ This study focused on authors experiences when appling simultaneous sural and soleus flaps from one incision for soft tissue reconstruction in large soft tissue defect of the leg.

## MATERIALS AND METHODS

**Fig. 1:**
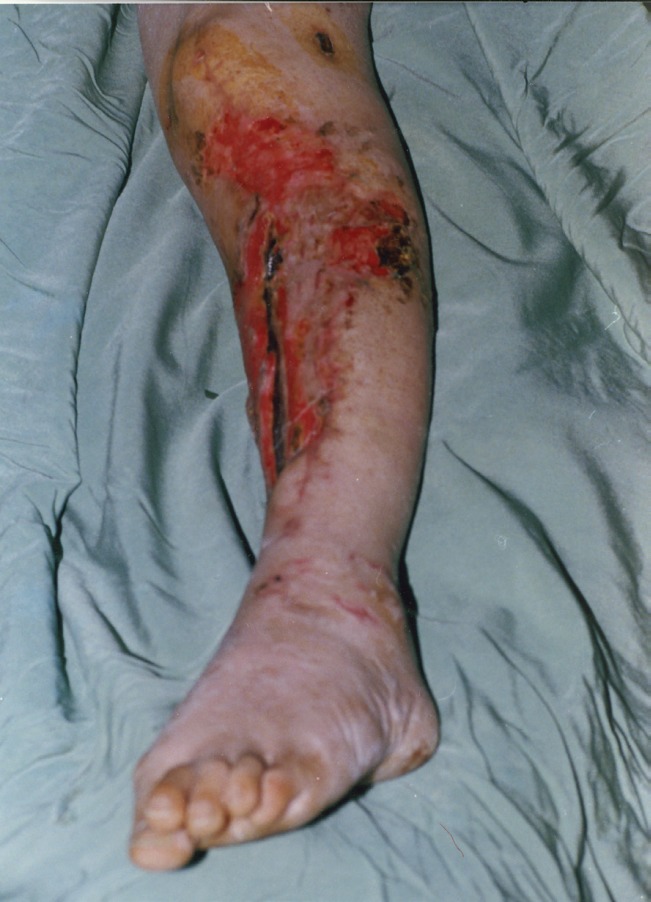
A 34-year-old man had large open tibial wound in the junction of the middle and distal thirds of the leg with an exposed tibial bone after debridement.

During 5 years period from 2009 to 2014, for 12 patients (10 males and 2 females) with large soft tissue defects in anterolateral of the leg, this procedure has been used. The patients’ age ranged from 22 to 58 years at the time of operation. Before operation, local soft tissue of lower limb around the defect was assessed carefully. Intact patients were selected for this method of surgery. Bone condition was evaluated and if needed, it was stabilized before soft tissue coverage. Surgical repair was performed under general anesthesia (Figure 1).

**Fig. 2:**
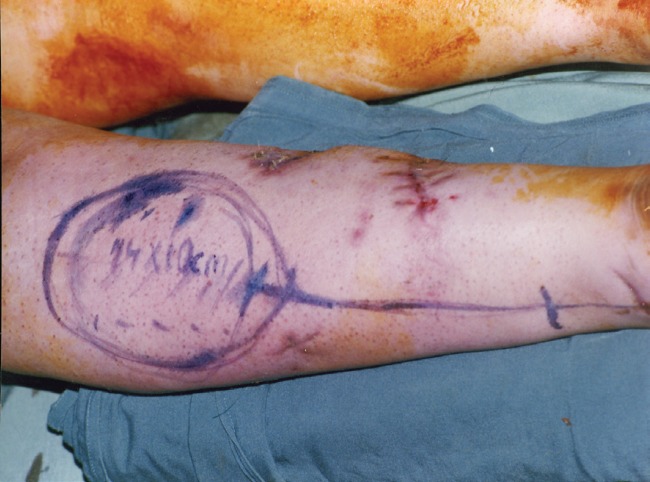
Marking of two flaps.

**Fig. 3:**
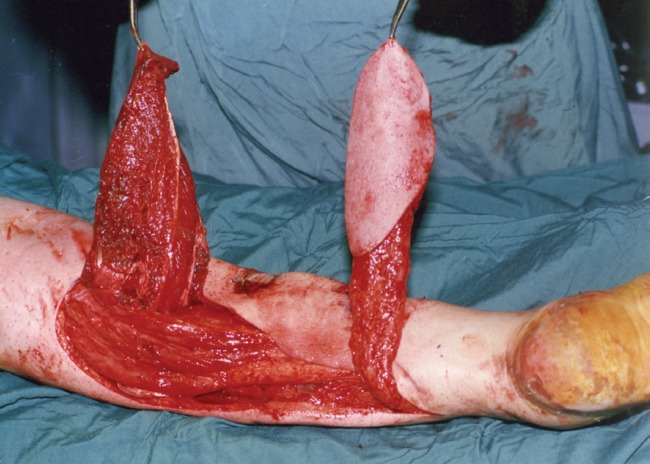
Intraoperative view after harvesting two flaps.

**Fig. 4:**
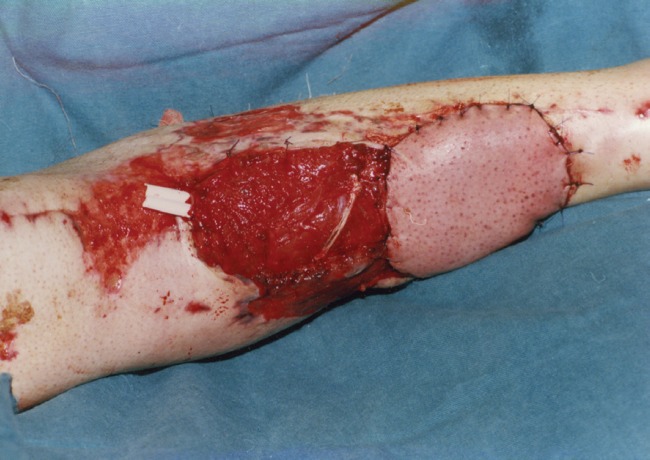
Coverage of defect with a combined soleus and sural muscle flap.

The method involved was marking the sural flap with maximum soft tissue that we can harvest. Total belly of soleus and reverse sural were approached from longitudinal lateral incision (Figures 2-4). The surgery was started by observing the antiseptic and aseptic techniques, with preparation of the entire lower limb in which the muscle transposition would take place, and also the contralateral thigh, when we wanted to use skin graft in the same procedure. After lifting limb for a few minutes, ischemia was made using a crepe bandage or Smarch tape, by inflating the cuff with a mean pressure of 100 mmHg above the blood systolic pressure.

## RESULTS

In these series, all patients were managed initially by the orthopedic trauma services. Their wounds were debrided by the primary service, then referred to the plastic surgery department. An open tibial wound in the junction of the middle and distal thirds of the leg was reconstructed successfully with the synchronous regional muscle and fasciocutaneus flaps. Twelve patients had primary healing of their tibial wounds were without any complications. 

Only one patient developed insignificant distal flap necrosis and was treated subsequently with surgical debridement and flap readvancement. His tibial wound healed completely after reoperation. All patients had reliable healing of their tibial wounds and evidenced healing of their fractures, with good cosmetic outcome following flap reconstructions. Limb salvage in those patients was also achieved during follow-up. Patients were able to ambulate postoperatively as instructed by the physiotherapist.

## DISCUSSION

During the last 2 decades since free tissue transfer became a standard surgical procedure for limb salvage, the outcome for surgical management of complex lower extremity wounds has improved dramatically.^1^^-^^4^ Although microsurgical flaps have been the method of choice for this reconstruction, many hospitals do not have equipment or microsurgical staff trained for this type of procedure and also an experienced surgeon is not available. Moreover, the patient’s clinical condition does not allow a more complex surgery in some cases. 

The management of the mid-third tibial wound can be challenging. If the defect is not too extensive, a soleus muscle flap such as medial hemisoleus muscle flap can be used successfully to cover such a wound.^5^ However, if the soleus muscle is traumatized, a microvascular free tissue transfer should be used to cover such a wound. In addition, a combined medial gastrocnemius and medial hemisoleus muscle flaps can also be used to cover a relatively large or extensive mid third tibial wound with good success.^6^

The distally based (or reverse) sural artery fasciocutaneous flap has gained much of the attention recently.^7^ The flap is found to be reliable and versatile and can be elevated quickly to cover a wound in the distal third of the leg. At the present time, the proper management of soft tissue coverage for a less extensive tibial wound in the junction of the middle and distal thirds of the leg has rarely been discussed in literature and therefore, the optimal reconstruction for this unique clinical problem has not been determined.^8^

A soleus muscle flap can be considered as an option. Based on the previous studies, the authors used the soleus muscle flap for reconstruction of an open tibial wound in the junction of the middle thirds of the leg and reverse sural with good success. For reconstruction of a wound in the distal third of the leg, a microvascular free tissue transfer has been considered as a standard surgical procedure of the choice because in general, there are no reliable local options available for reconstruction.^1^^,^^3^

In general, a microvascular free tissue transfer should still be considered for a larger soft-tissue wound in the distal third of the leg, or for a less extensive wound, when either the soleus muscle or those minor pedicles from the posterior tibial vessels are traumatized.^9^^,^^10^ The distally based (or reverse) sural artery fasciocutaneous flap has gained much of the attention recently.^7^ The flap is found to be reliable and versatile and can be elevated quickly to cover a wound in the distal third of the leg. It can even reach the foot and ankle area to cover both medial and lateral malleoli wounds and also a heal wound.^7^ The reverse soleus flap had an excellent outcome with short surgical duration, easy implementation, excellent resolution, and low morbidity of the donor area.

Occasionally, the venous congestion can be a problem for the flap and thus either surgical delay or supercharge of the flap has been recommended.^11^ Some other reverse adipose-fascial flaps can also be used to cover a wound in the distal leg.^12^ Other advantages of this procedure are in risk factor existence. These risk factors could impair successful defect coverage such as diabetes mellitus, and peripheral vascular disease. The aim of this method is to present an alternative option for reconstruction of large defect with combination of fasciocutaneous and muscular flap instead of free flap. Although, the entire soleus muscle used as a flap has a limited arc of rotation.^12^

Therefore, it may not reach a relatively distal soft tissue defect over the tibia. Limitation of plantar flexion of the foot may be another disadvantage, when the whole muscle is sacrificed. More importantly, the distal portion of the soleus muscle after elevation has traditionally been considered unreliable for coverage of a relatively distal tibial wound because of the muscle’s circulation pattern, but for proximal defect it is versatile flap.^12^

Soft tissue reconstruction of the middle and distal third of the leg represents a challenge for many plastic surgeons. Although microsurgical flaps have been the method of choice for this reconstruction, many hospitals do not have equipment or microsurgical staff trained for this type of procedure. Moreover, the patient’s clinical condition does not allow a more complex surgery in some cases. Based on the authors’ experience, the flap is reliable local option and can provide just enough muscle bulk for soft-tissue coverage of the extensive tibial soft tissue defect. The reconstructive outcomes are usually quite good and a low cost-effective approach. Also it can be performed by most reconstructive surgeons in selected patients.
